# Endometrial Inflammation at the Time of Insemination and Its Effect on Subsequent Fertility of Dairy Cows

**DOI:** 10.3390/ani11071858

**Published:** 2021-06-22

**Authors:** Karen Wagener, Marc Drillich, Christine Aurich, Christoph Gabler

**Affiliations:** 1 Department of Farm Animals and Veterinary Public Health, Clinical Unit for Herd Health Management in Ruminants, University Clinic for Ruminants, University of Veterinary Medicine Vienna, 1210 Vienna, Austria; Marc.Drillich@vetmeduni.ac.at; 2 Department for Small Animals and Horses, Centre for Artificial Insemination and Embryo Transfer, University of Veterinary Medicine Vienna, 1210 Vienna, Austria; Christine.Aurich@vetmeduni.ac.at; 3 Department of Veterinary Medicine, Freie Universität Berlin, Institute of Veterinary Biochemistry, 4163 Berlin, Germany; Christoph.Gabler@fu-berlin.de

**Keywords:** mRNA expression, PMN, endometrium, subfertility, cattle, dairy cow

## Abstract

**Simple Summary:**

A detailed understanding of cellular and molecular mechanisms in the bovine uterus is crucial to explain and avoid subfertility in dairy cows. Therefore, we examined the effect of inflammation in the bovine uterus in cows with no clinical signs of disease at the time of artificial insemination (AI) on subsequent pregnancy outcome. In a total of 71 healthy dairy cows, uterine cytology samples were collected by cytobrush technique within 10 min after insemination. Endometrial inflammation was investigated at the cellular and mRNA expression levels. All factors with a significant effect on fertility in our study were related to uterine polymorphonuclear neutrophil (PMN) migration, i.e., the first line of uterine defense. Cows with a proportion of ≥1% PMN had a 1.8-fold increased chance of pregnancy within 150 days postpartum compared to cows with fewer PMNs. From our results, we conclude that a certain level of inflammation at the molecular and cellular levels before the stimulus of AI might be favorable for cows’ fertility.

**Abstract:**

Our objective was to investigate the level of endometrial immune response at artificial insemination (AI) and to relate it to subsequent fertility. From 71 healthy cows, endometrial cytobrush samples were taken at the first AI for cytological and mRNA analyses. Total RNA isolated from the cytobrushes was used for reverse transcription qPCR for selected transcripts. Animals were grouped into pregnant (PREG; *n* = 32) and non-pregnant (non-PREG; *n* = 39) cows following their first AI. The mRNA abundance of the neutrophil-related factor *CEACAM1* and the chemokine *CXCL5* was 1.2- (*p* = 0.03) and 2.0-fold (*p* = 0.04) greater in PREG than in non-PREG cows, respectively. Animals were further subdivided according to the number of inseminations until pregnancy (PREG1, *n* = 32; PREG2-3, *n* = 19) and in repeat breeder cows (RBC, *n* = 13). *CEACAM1* and *CXCL8* mRNA expression was 1.7- (*p* = 0.01) and 2.3-fold (*p* = 0.03) greater in PREG1 than in RBC, respectively. Cox regression showed that cows with PMN ≥ 1% had a 1.8-fold increased chance of pregnancy within 150 days postpartum compared with cows with fewer PMNs. We conclude that a certain level of inflammation before the stimulus of AI might be beneficial for subsequent fertility.

## 1. Introduction

Subfertility represents a substantial problem for dairy cattle farming because the associated prolonged calving intervals, reduced milk yield and increased herd culling rates contribute to enormous economic losses [1]. The reasons for subfertility are manifold and complex. Besides ovarian cyclicity, efficient estrus detection and good breeding management, uterine health is key for the excellent reproductive performance of dairy herds [2,3]. To explore the underlying reasons for impaired uterine health, many studies have analyzed endometrial infection and inflammation in the postpartum period in cows with uterine diseases, such as clinical endometritis (CE) and subclinical endometritis (SE) [4,5,6,7,8,9].

During the first weeks after calving, tremendous morphological, immunological and microbial changes occur in the bovine uterus. Precise modulation of the maternal immune response is a prerequisite for uterine health and fertility [10]. Endometrial inflammation, characterized at the molecular level by a greater mRNA abundance of pro-inflammatory factors [4,5] and at the cellular level by a PMN influx [11], is the first line of uterine defense against uterine contaminants, pathogens and potential pathogens [12]. Inflammatory processes that persist beyond the voluntary waiting period might impair fertility by interfering with embryo development [13]. In cows with CE and SE, the uterine environment is well described and compared with healthy cows. At the day of diagnosis (days 24–30 postpartum (pp)), cows with CE or SE showed an increased abundance of transcripts of the pro-inflammatory factors interleukin (*IL*) *1A*, *IL1B*, *IL6*, chemokine CXL ligand (*CXCL*) *1/2*, *CXCL3*, *CXCL5* and *CXCL8* compared to healthy cows [4,5]. A considerably high percentage of animals without CE or SE, however, also fail to become pregnant [14]. A detailed understanding of the cellular and molecular mechanisms in the bovine uterus in healthy and diseased cows is crucial to explain and to avoid subfertility in cows with and without apparent uterine health problems. In healthy cows, a certain base level of mRNA expression of pro-inflammatory factors exists [4,5]. However, little is known about the association between mRNA expression of these factors and fertility.

To examine the reason for subfertility in clinically healthy cows, studies have focused on repeat breeder cows (RBCs), i.e., cyclic cows that fail to conceive after at least three subsequent inseminations without showing clinical abnormalities [7,15,16,17,18]. It has been demonstrated that RBCs without SE showed a greater mRNA expression of pro-inflammatory factors and mucins compared to a healthy and fertile control group [7,17]. RBCs, however, are a heterogeneous group of animals in terms of days in milk (DIM), as the identification of RBCs is based on the time of the third unsuccessful insemination, which strongly depends on the breeding management of the farms. Therefore, in the present study, we focus on the time of the first artificial insemination (AI) after calving to characterize the endometrial inflammatory state within a narrow timeframe and to relate the findings to subsequent fertility.

There is evidence that an upregulated cellular immune response in the uterus after AI is advantageous for subsequent fertility [13,19]. Moderate PMN infiltration 4 h after AI increased the likelihood for pregnancy [19]. In addition, a PMN% increase from AI to embryo collection 7 days later increased the number of transferable embryos in superovulated cows [13]. The observed phenomenon in the latter studies could be interpreted as a physiological post-breeding inflammation. Thus far, little is known with regard to the molecular mechanisms accompanying the cellular immune response of PMN infiltration at the time of AI.

A targeted analysis of transcripts revealed a physiologically upregulated mRNA expression of *CXCL5*, *IL1B* and *CXCL8* during estrus [5]. A transcriptome analysis showed upregulated mRNA expression of genes related to extracellular matrix remodeling, transport and cell growth and morphogenesis during estrus, with no differences between early and late estrus [20]. In both studies, animals were slaughtered before sampling, and thus, the impact of this upregulated immune response for fertility remains elusive.

Therefore, the objective of the current study was to investigate the level of uterine PMN% and endometrial mRNA expression of selected pro-inflammatory factors and mucins at the time of the first AI after calving and to relate the level of inflammation to the subsequent fertility. Our main hypothesis was that cows conceiving from their first AI present greater mRNA expression levels of pro-inflammatory factors and mucins and greater endometrial PMN% at the time of AI than their non-pregnant counterparts do.

## 2. Materials and Methods

### 2.1. Study Farm and Reproductive Management

The study was approved by the Slovakian Regional Veterinary Food Administration and by the institutional ethics committee of the University of Veterinary Medicine Vienna (ETK-01/12/2015).

The study was performed between April 2015 and March 2016 on a commercial dairy farm in Slovakia housing 2400 Holstein-Friesian cows. The average energy-corrected milk yield was 9,120 kg. Animals were housed in free-stall barns with high-bed cubicles. During the close-up and at the end of the pp period (60 ± 10 days pp), the body condition score (BCS) was assessed on a 5-point scale [21]. On day 5 ± 2 pp, whole blood samples were taken from the coccygeal vein for beta-hydroxybutyrate (BHB) measurement using an electronic handheld device (FreeStyle Precision, Abbott, Wiesbaden, Germany). Cows with BHB ≥ 1.2 mmol/L were defined as having subclinical ketosis and received 250 mL of propylene glycol orally for 5 consecutive days.

On days 5 ± 3 and 32 ± 2 pp, a gynecological examination was performed by transrectal palpation and vaginal examination with a gloved hand. Puerperal metritis was defined as foul smelling, reddish-brown vaginal discharge and a rectal temperature >39.5 °C [22] on day 5 ± 3 pp. Affected cows were systemically treated with ceftiofur for 3 to 5 days.

Cows with vaginal mucus with flecks of pus, mucopurulent or purulent discharge on day 32 ± 2 pp were defined as having CE [22] and were treated with the prostaglandin F2α (PGF2α)-analogue dinoprost trometamol twice 14 days apart. The voluntary waiting period was set at 50 days pp.

For heat detection, visual observation was combined with an automated heat detection system (CowManager Sensoor, Agis, Harmelen, The Netherlands). All animals not detected in estrus until day 70 pp were submitted to an Ovsynch protocol with fixed-time AI [23] and were included in the study. On day 39 ± 3 after AI, pregnancy checks were performed by ultrasound. Animals seen in estrus before pregnancy diagnosis were re-inseminated. All the other animals were included in a Resynch protocol 7 days before the first pregnancy diagnosis. In non-pregnant cows, the Resynch protocol with fixed-time AI was continued [24].

### 2.2. Study Population

Only cows receiving their first AI after initiation of the Ovsynch protocol were included in the study. We decided to include only cows subjected to an Ovsynch protocol with fixed-time AI to avoid any bias due to individual decisions made by the AI technicians. Another inclusion criterion was the absence of any signs of clinical endometritis at AI. Therefore, the vaginal discharge was evaluated with a Metricheck device (Simcro, Hamilton, New Zealand) directly after AI as previously described [25] to select cows with clear vaginal discharge (*n* = 74).

Downer cows, i.e., animals with a history of clinical ketosis or clinical hypocalcemia during the current lactation, and cows with an abortion were not included in the analyses. Thus, all successful inseminations ended up with successful calving. To exclude cows not responding to the Ovsynch protocol, the progesterone concentration (P4) was determined in blood plasma at the time of AI, i.e., 16–18 h after the second GnRH application. For this purpose, blood samples were taken from the coccygeal vein (Lithium Heparin Vacuette tubes, Greiner bio-one, Kremsmünster, Austria) and P4 concentrations were determined by a validated enzyme-linked immunosorbent assay (Progesterone ELISA kit, Enzo Life Sciences, Farmingdale, NY, USA). The intra-assay coefficient of variation was 9.4% and the inter-assay coefficient of variation was 15.4% [17]. Three cows with P4 > 1 ng/mL were excluded from the study. Thus, the final study population comprised 71 animals.

To test our primary hypothesis that cows conceiving from their first AI show a greater level of uterine inflammation than cows not getting pregnant from the first AI do, we grouped animals into pregnant (PREG; *n* = 32) and non-pregnant (non-PREG; *n* = 39) cows following their first AI. The study animals were further subdivided according to the number of inseminations until 150 DIM: animals in the PREG group that conceived after the first AI were designated as PREG1 (*n* = 32). Animals in the non-PREG group were subdivided into cows conceiving after the second or third AI (PREG2-3; *n* = 19) and into animals not pregnant after the third AI, referred to as RBCs (*n* = 13). Seven cows were removed from this last subdivision step because they were excluded from breeding after the first (*n* = 1) or second (*n* = 6) AI because of clinical or subclinical mastitis.

### 2.3. Endometrial Sampling, Cytological and mRNA Analyses

Endometrial sampling was performed with the cytobrush technique within the first 10 min after the first AI after calving as described by Wagener et al. [17]. Sampling was not repeated during the following AIs. The farm management did not allow to collect samples shortly before or simultaneously with AI. It is well-known that cytobrush sampling at AI has no negative effect on subsequent fertility [19]. Directly after sampling, the cytobrush was rolled on a disinfected microscope slide to determine the proportion of PMNs in the sample as described in detail by Prunner et al. [26]. Subsequently, the cytobrush was transferred into a cryotube, which was placed within 5 to 10 min into liquid nitrogen and stored at –80 °C until mRNA analyses.

The total RNA was extracted from the cytobrushes using the RNeasy Plus Mini Kit (Qiagen, Hilden, Germany) as described in detail by Odau et al. [27]. The concentration of the isolated total RNA was determined by spectrophotometry at a wavelength of 260 nm (NanoDrop ND-1000, Peqlab Biotechnology, Erlangen, Germany). For quality assessment, the RNA integrity was determined with a Bioanalyzer 2100 using the Agilent RNA 6000 Nano Kit (both Agilent Technologies, Waldbronn, Germany). To remove possible contamination with genomic DNA, a DNase digestion step was carried out before reverse transcription. Reverse transcription was performed with 500 ng total RNA by adding 200 U RevertAid reverse transcriptase, 2.5 µM random hexamers and 0.67 mM each of dNTP and 1× reverse transcriptase buffer (all Thermo Scientific, Langenselbold, Germany) in a total volume of 60 µL as previously described [17]. To monitor potential genomic DNA or contaminations, negative controls without reverse transcriptase were generated.

The qPCR was carried out with the StepOne Plus cycler (Applied Biosystems, Darmstadt, Germany) following the minimum information for publication of quantitative real-time PCR experiments (MIQE) guidelines [28]. The selected pro-inflammatory factors were IL1A, IL1B, prostaglandin-endoperoxide synthase 2 (PTGS2), carcinoembryonic antigen-related cell adhesion molecules (CEACAM, formerly known as CD66), CXCL3, CXCL5, CXCL8, tracheal antimicrobial peptide (TAP) and the mucins (MUC) MUC4 and MUC16. Amplification was performed with a reaction mixture containing 1 µL cDNA, 0.4 µM of each primer (Table 1) and 1× SensiMix SYBR Low-ROX (Bioline, Luckenwalde, Germany) in a total volume of 10 µL. The qPCR was performed after 10 min incubation at 95 °C with 45 cycling repeats of the following protocol: 15 s denaturation at 95 °C, an annealing step for 20 s at indicated annealing temperatures (Table 1) and elongation at 72 °C for 30 s. A melting curve with continuous fluorescence monitoring was performed to verify specific cDNA amplification. Standard curves of specific PCR products with known concentrations were used to determine the contents of specific mRNA [17].

For normalization, five potential reference genes were initially assessed: B-Aktin (ACTB), glyceraldehyde 3-phosphate dehydrogenase (GAPDH), ribosomal protein L19 (RPL19), succinate dehydrogenase complex subunit A (SDHA) and suppressor of zeste 12 homolog (SUZ12). The geNorm software [29] was used to identify the most stably expressed genes. *SUZ12* and *RPL19* were finally used to calculate the normalization factor.

**Table 1:** Primer sequences, resulting amplicon length, annealing temperatures used for quantitative PCR for selected transcripts and reference genes.

Gene	Nucleotide Sequence	Amplicon Length (bp)	Annealing Temp. (°C)	Reference/Accession No.
*IL1A*	F: 5′-TCA TCC ACC AGG AAT GCA TC-3′	300	59	NM_174092
	R: 5′-AGC CAT GCT TTT CCC AGA AG-3′			
*IL1B*	F: 5′-CAA GGA GAG GAA AGA GAC A-3′	236	56	[30]
	R: 5′-TGA GAA GTG CTG ATG TAC CA-3′			
*CEACAM1*	F: 5′-CCC AGA ACA CCT CCT ACA TG 3′	336	58	AY345130
	R: 5′-TCT GTG CAA GGA GGA GAC TC 3′			
*CXCL3*	F: 5′-GCC ATT GCC TGC AAA CTT-3′	189	56	[30]
	R: 5′-TGC TGC CCT TGT TTA GCA-3′			
*CXCL5*	F: 5′-TGA GAC TGC TAT CCA GCC G-3′	193	61	[17]
	R: 5′-AGA TCA CTG ACC GTT TTG GG-3′			
*CXCL8*	F: 5′-CGA TGC CAA TGC ATA AAA AC-3′	153	56	[30]
	R: 5′-CTT TTC CTT GGG GTT TAG GC-3′			
*PTGS2*	F: 5′-CTC TTC CTC CTG TGC CTG AT-3′	359	60	[30]
	R: 5′-CTG AGT ATC TTT GAC TGT GGG AG-3′			
*TAP*	F: 5′-CTC TTC CTG GTC CTG TC-3′	184	60	[17]
	R: 5′-GCT GTG TCT TGG CCT TCT TT-3′			
*MUC4*	F: 5′-ACG TCA CTG TGC ATC TTT GG-3′	199	60	[17]
	R: 5′-AAG CTC TTG ATG GAC GGT TG-3′			
*MUC16*	F: 5′-CAG GTC TCA AAA TCC CAT CC-3′	256	62	[17]
	R: 5′-TGC TGG AGG TGT TGA TAT GG-3′			
*ACTB*	F: 5′-CGG TGC CCA TCT ATG AGG-3′	266	58	AY141970
	R: 5′-GAT GGT GAT GAC CTG CCC-3′			
*GAPDH*	F: 5′-GAA GGT GAA GGT CGG AGT CAA C-3′	306	62	[31]
	R: 5′-CAG AGT TAA AAG CAG CCC TGG T -3′			
*RPL19*	F: 5′-GGC AGG CAT ATG GGT ATA GG- 3′	232	60	NM_001040516.1
	R: 5′-CCT TGT CTG CCT TCA GCT TG- 3′			
*SDHA*	F: 5′-GGG AGG ACT TCA AGG AGA GG-3′	291	60	[30]
	R: 5′-CTC CTC AGT AGG AGC GGA TG-3′			
*SUZ12*	F: 5′-GAA CAC CTA TCA CAC ACA TTC TTG T-3′	359	60	[17]
	R: 5′-TAG AGG CGG TTG TGT CCA CT-3′			

### 2.4. Statistical Analyses

Data management and statistical analysis were carried out with Microsoft Excel (Excel 2010, Microsoft Office, Redmond, WA, USA) and SPSS (version 26.0, IBM SPSS, Munich, Germany). For visualization of the mRNA expression, GraphPad Prism version 7.00 software (GraphPad Software, La Jolla, CA, USA) was used. According to the Kolmogorov–Smirnov and Shapiro–Wilk tests, data were not normally distributed. Sample size calculation was performed using the G*Power software (version 3.1.9.2, Heinrich-Heine-University Düsseldorf, Düsseldorf, Germany). The effect size was calculated on the basis of previously published data [17]. Sample size calculations with an effect size of 0.64, α = 0.05 and power = 0.8 revealed that a total sample size of 30 animals per group was needed to test the hypothesis on differential mRNA expression between PREG (*n* = 32) and non-PREG (*n* = 39) using the Mann–Whitney U test.

Comparisons between PREG1 (*n* = 32), PREG2-3 (*n* = 19) and RBC (*n* = 13) were performed using the non-parametric Kruskal–Wallis test with pairwise multiple comparisons. Removed cows (*n* = 7) were not included in this analysis. The univariate chi-squared test was used to analyze the effect of parity, BCS loss and postpartum diseases on the first service conception rate. The odds of pregnancy following the first AI were analyzed by a binary logistic regression model. Included covariates were parity (1st = 0 (reference), 2nd = 1, ≥3rd = 2), BCS loss during the first 60 days pp (<1 BCS-point = 0, ≥1 BCS-point = 1), history of subclinical ketosis day 5 ± 2 pp, puerperal metritis day 5 ± 3 pp, CE day 34 ± 7 pp (0 = no, 1 = yes) and normalized mRNA expression of selected pro-inflammatory factors and mucins (metrically scaled). Covariates with a *p*-value > 0.1 were stepwise removed from the models in descending order (backward elimination). For the remaining variables, odds ratios (ORs) were calculated with 95% CI. With the same covariates and settings, a Cox model was used to calculate the chance of conceiving within 150 DIM. A Kaplan–Meier survival analysis for the proportion of non-pregnant cows was calculated from enrollment (day 0) until 150 days after enrollment for cows with PMN ≥ 1% and PMN < 1%. Removed cows (*n* = 7) were also excluded from the survival analyses. Values are expressed in the text as mean ± standard deviation (SD). The normalized mRNA expression is presented as box-and-whisker plots with median values and 50% of the data within the boxes. Whiskers include all data points within 1.5 times the interquartile range (IQR). All outliers (≥1.5 times IQR) were included in the statistical analyses. The level of significance was set at *p* ≤ 0.05, and a *p*-value above this threshold but <0.1 was regarded as a trend towards statistical significance.

## 3. Results

### 3.1. Study Population

Most of the animals were in their first (36.6%) or second (35.2%) lactation and the remaining cows had >2 lactations. At enrollment, i.e., the first AI after calving, animals were 83.7 ± 3.4 SD days in milk. Between the close-up period and 90 days pp, a BCS loss of ≥ 1 BCS-point was observed in 46.5% of the animals, and in the remaining animals, the BCS loss was lower than 1 BCS-point. Subclinical ketosis, puerperal metritis and CE were diagnosed in 12.7%, 16.9% and 18.3% of the cows, respectively. The univariate analysis revealed no significant effect of parity, BCS loss and recorded postpartum diseases on the first service conception rate (Table 2).

### 3.2. Effect of Endometrial Neutrophil Infiltration and mRNA Expression on the First Service Conception Rate

The mean PMN percentage in PREG and non-PREG was 0.9 ± 1.3% and 0.9 ± 1.2% SD (*p* > 0.05), respectively, and none of the animals exceeded a PMN threshold of 5%. The threshold of 1% PMN, suggested by Pascottini et al. [32] for the diagnosis of subclinical endometritis at AI, was exceeded by 39.4% (28/71) of the cows. The percentage of cows with PMN ≥ 1% was 46.9% (15/32) in PREG and 33.3% (13/39) in non-PREG (*p* > 0.05). The first service conception rate did not differ significantly between cows with PMN ≥ 1% (53.6%; 15/28) and < 1% (39.5%; 17/43).

**Figure 1:**
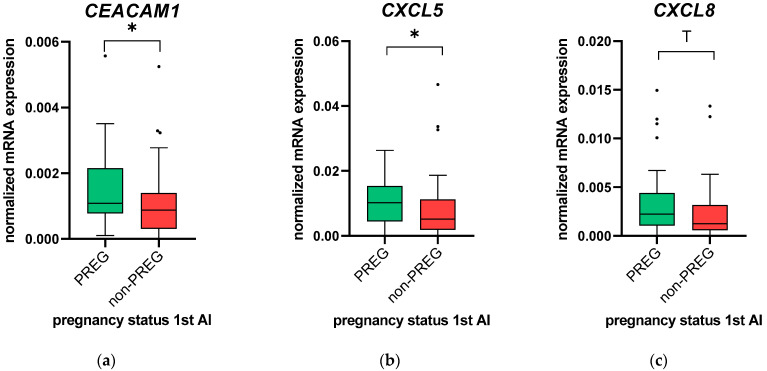
Normalized *CEACAM1* (**a**), *CXCL5* (**b**) and *CXCL8* (**c**) mRNA expression in endometrial cytobrush samples collected from animals at the time of artificial insemination (AI). Animals were grouped by animals that conceived (PREG; *n* = 32) or did not conceive (non-PREG; *n* = 39) from the first insemination after calving. Values are represented as box-and-whisker plots with median values and 50% of the data within the boxes. Whiskers include all data points within 1.5 times the interquartile range (IQR). All outliers (≥1.5 times IQR) were included in the statistical analyses. Significant differences with *p* ≤ 0.05 between groups are indicated with an asterisk (*), and a trend towards statistical significance (*p* = 0.051–0.1) is marked with a “T”.

Figure 1 shows the level of mRNA expression of *CEACAM1* (a), *CXCL5* (b) and *CXCL8* (c) in PREG and non-PREG. The mRNA expression of the neutrophil-related factor *CEACAM1* was higher in PREG than in non-PREG (1.2-fold; *p* = 0.03). The chemokine *CXCL5* expression was 2.0-fold higher in PREG than in non-PREG (*p* = 0.04), and for the chemokine *CXCL8*, a trend towards significantly higher mRNA expression in PREG was observed compared with non-PREG (1.8-fold; *p* = 0.11). For all the other pro-inflammatory factors and mucins, no significant differences in the mRNA expression were found between PREG and non-PREG (Figure S1).

**Figure 2:**
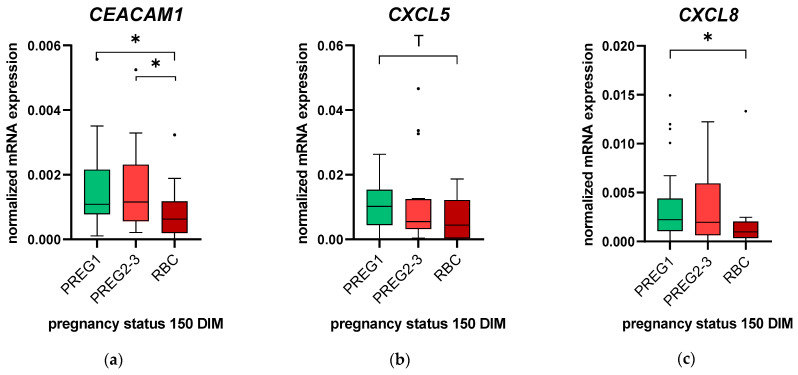
Normalized *CEACAM1* (**a**), *CXCL5* (**b**) and *CXCL8* (**c**) mRNA expression in endometrial cytobrush samples collected from animals at the time of artificial insemination. Animals were retrospectively grouped by animals that conceived after the first (PREG1, *n* = 32) or second or third AI (PREG2-3, *n* = 19) or animals with more than three unsuccessful AIs, i.e., repeat breeder cows (RBC, *n* = 13), until 150 days in milk (DIM). Values are represented as box-and-whisker plots with median values and 50% of the data within the boxes. Whiskers include all data points within 1.5 times the interquartile range (IQR). All outliers (≥1.5 times IQR) were included in the statistical analyses. Significant differences with *p* ≤ 0.05 between groups are indicated with an asterisk (*), and a trend towards statistical significance (*p* = 0.051–0.1) is marked with a “T”.

In the next step, the groups PREG1, PREG2-3 and RBC were analyzed (Figure 2). The Kruskal–Wallis test revealed an association between groups and the level of *CEACAM1* mRNA expression (*p* = 0.03) and a tendency towards significance for *CXCL8* (*p* = 0.09). *CXCL5* and the other selected factors were not related to the groups (*p* > 0.1). After pairwise multiple comparison, the most striking differences in the endometrial mRNA expression were found between PREG1 and RBC. *CEACAM1* and *CXCL8* mRNA expression was 1.7- (*p* = 0.01) and 2.3-fold (*p* = 0.03) higher in PREG1 compared with RBC, and PREG1 tended to have greater (2.3-fold) *CXCL5* mRNA abundance than RBC did (*p* = 0.08). For *CEACAM1*, differences were also found between PREG1, PREG2-3 and RBC (*p* = 0.03). For all the other pro-inflammatory factors and mucins, no significant differences in the mRNA expression were found between groups (Figure S2).

In the backwards binomial regression analysis, *CEACAM1* mRNA expression was the only remaining factor with a tendency towards a significant effect on the first service conception rate (OR = 3.8 × 10^156^; CI = 0.06–7.2 × 10^307^; *p* = 0.052).

### 3.3. Effect of Endometrial Neutrophil Infiltration and mRNA Expression on the Time to Pregnancy

**Figure 3:**
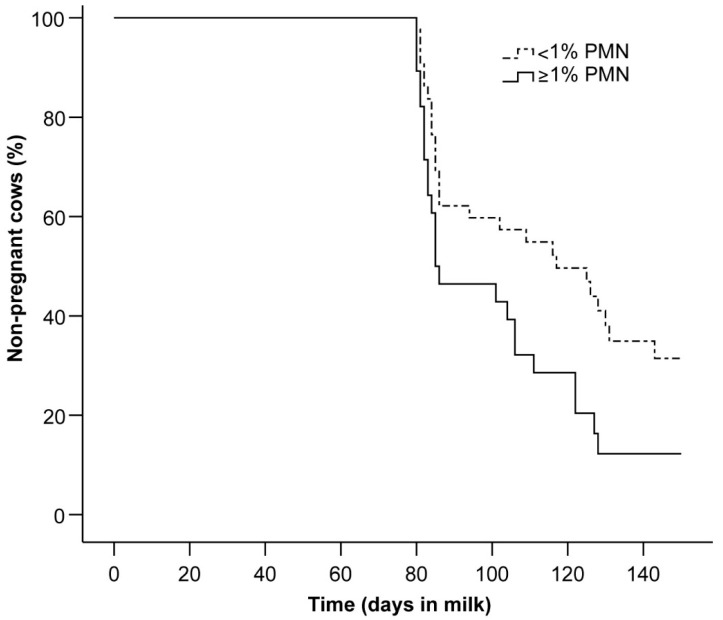
Kaplan–Meier survival curves for the percentage of non-pregnant cows until 150 days in milk for animals with PMN ≥ 1% or PMN < 1%. The percentage of cows removed from the analysis because they were excluded from breeding after the first (*n* = 1) or second (*n* = 6) AI was 3.6% (1/28) for PMN ≥ 1% and 14% (6/43) for PMN < 1%.

The Cox proportional hazard analysis showed that the endometrial PMN percentage and *MUC16* mRNA expression were the only remaining factors with effects on the time to pregnancy. Cows with PMN ≥ 1% had a 1.8-fold increased chance of pregnancy within 150 days pp compared with cows with PMN < 1% (hazard ratio = 1.8; 95% CI = 1.0 to 3.2; *p* = 0.05), and *MUC16* mRNA expression was also significantly related to the time to pregnancy (hazard ratio = 6.2; 95% CI = 1.6 to 23.9; *p* = 0.008). Figure 3 presents the survival curve for the time to pregnancy for cows with PMN ≥1% or <1%.

## 4. Discussion

There is a wealth of information with regard to the presence of uterine inflammation in cows with CE and SE [4,5], but only a few studies have examined the level of the endometrial immune response in healthy cows at AI and the effect on subsequent fertility. There are hints in the literature that moderate inflammation after AI could be advantageous for fertility [13,19]. Therefore, in our study, we tested the hypothesis that cows conceiving from their first AI show a greater level of uterine inflammation than cows not getting pregnant from the first AI.

From all investigated factors, endometrial mRNA expression of the chemokines *CXCL5* and *CXCL8* and *CEACAM1* and uterine PMN infiltration were positively related to the subsequent pregnancy outcome. Thus, the results confirm our hypothesis and indicate that a certain level of inflammation at AI may be needed for successful insemination. All factors with a significant effect on fertility in our study have in common the fact that they are related to PMN migration into the uterus [33]. The chemokines *CXCL5* and *CXCL8* are involved in PMN recruitment [33] and *CEACAM1* mRNA encodes for a neutrophil adhesion molecule [34].

Our finding that cows with PMN ≥ 1% at AI had a 1.8-fold increased chance of becoming pregnant within 150 days pp compared with cows with fewer PMNs is in line with the findings of Drillich et al. [13] and Kaufmann et al. [19] showing that PMN infiltration after AI is beneficial for fertility. It is important to note, however, that in the cited studies, sampling was performed 4 h [19] and 7 days [13] after AI, whereas in our study, sampling was performed within 10 min after AI. The physiological meaning of increased PMN infiltration after AI is the removal of bacterial contamination and dead sperm [35]. It has been reported that PMN infiltration starts within 3 h following AI, peaks 6 h after AI and completely resolves 10 h after AI [35]. Therefore, it is very unlikely that the immune response in our study was induced by AI within the 10 min between AI and sampling. Other studies where sampling was performed simultaneously with the AI showed opposite results and demonstrated that cows exceeding a threshold of 1% PMN directly at AI have a lower chance to become pregnant from that insemination than cows with fewer PMNs do [16,32]. The main obvious differences between the cited studies and our study were the DIM at AI and, consequently, the DIM at sampling. In our study, sampling was performed at the first AI 83.7 ± 3.4 SD DIM, while in other studies, sampling was performed much later and cows were 122 ± 54 DIM [32] and 208.6 ± 96.2 DIM [16]. In addition, the mean proportion of PMNs in all cows at AI was generally lower in our study (0.9 ± 1.2% SD) compared to others (1.5 ± 5 SD [32]). In addition, we deliberately sampled the cows at their first AI to include a homogeneous group of animals. Since sampling was not repeated during the following AIs, there was a long time between the characterization of the uterine environment and the latest observation time of the study, which was 150 DIM. Thus, our findings from the survival analyses represent the long term-effects of the uterine environment at first AI on fertility. Although the study animals were closely monitored during the postpartum period, it cannot be excluded that other factors not considered in the study, such as heat stress or metabolic imbalances [36], had an influence on the reproductive performance of the cows.

Supported by other research, we come to the suggestion that the point at which inflammation switches from a physiological to a pathological state is not a black and white threshold phenomenon but depends on many factors, such as days postpartum and the level and duration of inflammation [4,37]. Therefore, a general threshold of 1% PMN, as suggested by Pascottini et al. [32] for the diagnosis of SE at AI, must be regarded critically.

From our results, we conclude that it appears favorable for the cow’s fertility if a certain amount of PMN is already present before the stimulus of AI. The fact that uterine sampling was performed directly after AI corroborates the theory that the observed uterine inflammation was not a post-breeding reaction but represents a physiologically upregulated immune response during estrus, which is regulated by estradiol [38].

It would be interesting to test in future studies, by repeated sampling before, during and after AI, whether the extent of breeding-induced endometrial inflammation depends on the onset of inflammation during estrus and if the ability to downregulate this inflammation during the luteal phase has an effect on fertility.

PMN infiltration represents only the cellular immune response. A better understanding of inflammatory mechanisms at the molecular level could open new avenues to manage and improve reproductive performance [5]. The mRNA abundance of the chemokines *CXCL8* and *CXCL5* at first AI pp was positively related to the pregnancy outcome in the current study. The function of *CXCL8* and *CXCL5* is the recruitment of PMN into the uterine lumen. Physiologically, the mRNA expression of these chemokines is upregulated during estrus [5] to modulate the above described PMN-related functions. While in general, progesterone has an immunosuppressive function, estradiol upregulates the uterine immune response, and both steroid hormones have a key role for uterine receptiveness [35,38]. We only measured the progesterone concentration on the day of AI to exclude animals not responding to the Ovsynch protocol. Since we did not monitor dynamic changes in the hormone status of the animals, we cannot answer the question of whether differences in the immune response or insemination success were caused by variations in the steroid hormone concentrations around the time of AI.

This is the first study showing that an increased chemokine mRNA expression at AI is favorable for subsequent fertility. It can be speculated that increased chemokine mRNA abundance during estrus reflects the preparation of the timely recruitment of PMN after breeding. The results demonstrate that it would be interesting to test in future studies whether uterine function can be restored in subfertile animals without clinical symptoms by using immunomodulators around breeding. The use of recombinant *CXCL8* as an immunomodulator constitutes a new attempt to treat and prevent uterine diseases [10,39]. It must be noted that our study was performed on a single farm with a limited number of animals. The results may not be representative for other farms since fertility strongly depends on farm-related factors, such as feeding and breeding management, as reviewed by Walsh et al. [2]. Therefore, further studies on different farms with a larger sample size are needed to confirm our results.

The most pronounced differences between cows conceiving from their first AI and cows with more than one AI were observed for *CEACAM1* mRNA expression. Little is known with regard to mRNA expression of *CEACAM1* in the bovine uterus. From studies in humans, we know that *CEACAM1* is an adhesion molecule for PMN [34], which is involved in the formation of neutrophil extracellular traps (NETs) [40]. NETs are aggregates of PMN and PMN-derived molecules that are involved in the defense against microorganisms [41]. NETs are also associated with uterine diseases and have been described in women with endometriosis [42] and mares with endometriosis [43]. Recently, NET formation was also detected in bovine endometrial tissue [44], but their physiological or pathological role in the bovine uterus is unknown. Our results justify further studies on *CEACAM1* and NET formation in the bovine endometrium to elucidate the role of this factor for bovine fertility.

Although our study demonstrates effects of an endometrial immune response at AI on subsequent fertility, our study cannot answer the question of whether altered endometrial cell function, pre-ovulatory estradiol concentration or metabolic imbalances lead to differential immune responses between animals. Our results provide a basis for future holistic studies on the dynamics of the inflammatory uterine environment, including hormonal, mRNA expression, cytological and protein analyses.

## 5. Conclusions

With our study, we suggest that upregulated mRNA expression of factors related with uterine PMN migration at AI positively affects fertility. Here, mRNA expression of the chemokines *CXCL8* and *CXCL5* and the PMN adhesion factor *CEACAM1* and PMN infiltration by itself were positively related to the pregnancy outcome. We conclude that a certain level of inflammation at the molecular and cellular levels at AI might be favorable for the cow’s fertility. The level and circumstances at which the inflammatory response switches from a physiological to a pathological state remain elusive. These results underline the need for detailed studies on the dynamics of the endometrial inflammatory response before, during and after AI and its implication for fertility.

## Figures and Tables

**Table 2:** Univariate analysis of the effect of lactation number, body condition score (BCS) loss and postpartum (pp) diseases on the first service conception rate of all cows (*n* = 71). The number of pregnant animals and the total number within the group are given in each category (*n*), and the percentage of pregnant animals (%) is shown.

Independent Variable	First Service Conception Rate		*p*-Value
	***n***	**%**	
Parity			0.26
1st	15/26	57.7	
2nd	9/25	36.0	
≥3rd	8/20	40.0	
BCS loss during the first 60 days pp ^1^			0.93
<1 BCS-point	16/36	44.4	
≥1 BCS-point	15/33	45.5	
Subclinical ketosis day 5 ± 2 pp			1.00
No	28/62	45.2	
Yes	4/9	44.4	
Puerperal metritis day 5 ± 3 pp			0.93
No	26/58	44.8	
Yes	6/13	46.2	
Clinical endometritis day 34 ± 7 pp ^1^			0.34
No	25/58	43.1	
Yes	7/12	58.3	

^1^ The sum of animals is not equal to 71 because of missing data for BCS (*n* = 2) and clinical endometritis (*n* = 1).

## Data Availability

Data are contained within the article or Supplementary Materials. Raw data are available on request from the corresponding author.

## Supplementary Materials

The following are available online at https://www.mdpi.com/article/10.3390/ani11071858/s1: Figure S1. Normalized *IL1A* (**a**), *IL1B* (**b**), *CXCL3* (**c**), *PTGS2* (**d**), *TAP* (**e**), *MUC4* (**f**) and *MUC16* (**g**) mRNA expression in endometrial cytobrush samples collected from animals at the time of artificial insemination (AI). Animals were grouped by animals that conceived (PREG; *n* = 32) or did not conceive (non-PREG; *n* = 39) from the first insemination after calving. Values are represented as box-and-whisker plots with median values and 50% of the data within the boxes. Whiskers include all data points within 1.5 times the interquartile range (IQR). All outliers (≥1.5 times IQR) were included in the statistical analyses. Figure S2. Normalized *IL1A* (**a**), *IL1B* (**b**), *CXCL3* (**c**), *PTGS2* (**d**), *TAP* (**e**), *MUC4* (**f**) and *MUC16* (**g**) mRNA expression in endometrial cytobrush samples collected from animals at the time of artificial insemination. Animals were retrospectively grouped by animals that conceived after the first (PREG1, *n* = 32) or second or third (PREG2-3, *n* = 19) AI or animals with more than three unsuccessful AIs, i.e., repeat breeder cows (RBC, *n* = 13), until 150 days in milk (DIM). Values are represented as box-and-whisker plots with median values and 50% of the data within the boxes. Whiskers include all data points within 1.5 times the interquartile range (IQR). All outliers (≥1.5 times IQR) were included in the statistical analyses.
