# Supplementary material for: Endometrial Inflammation at the Time of Insemination and Its Effect on Subsequent Fertility of Dairy Cows
Source: Animals (Basel). 2021 Jun 22;11(7):1858. doi: 10.3390/ani11071858 (PMC8300153; doi:10.3390/ani11071858)
Supplement: Supplementary file 1 [file animals-11-01858-s001.zip › animals-1258967-supplementary.pdf]

Figure S1

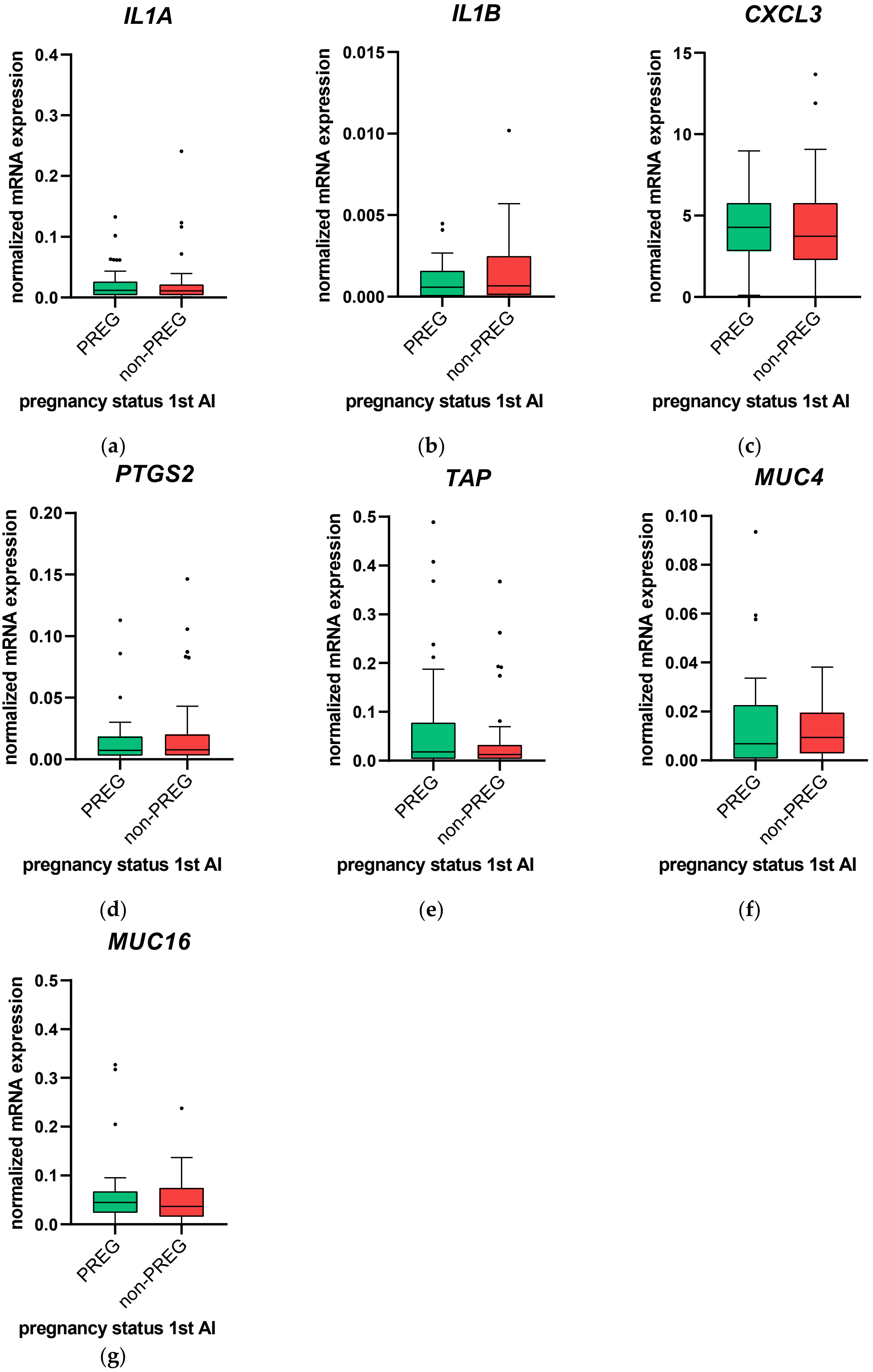

Figure S1. Normalized *IL1A* (**a**), *IL1B* (**b**) and *CXCL3* (**c**), *PTGS2* (**d**), *TAP* (**e**), *MUC4* (**f**), *MUC16* (**g**) mRNA expression in endometrial cytobrush samples collected from animals at the time of artificial insemination (AI). Animals were grouped in animals that conceived (PREG; *n* = 32) or did not conceive (non-PREG; *n* = 39) from the first insemination after calving. Values are represented as box and whisker plots with median values and 50% of the data within the boxes. Whiskers include all data points within 1.5 times the interquartile range (IQR). All outliers (≥1.5 times IQR) were included in the statistical analyses.

Figure S2

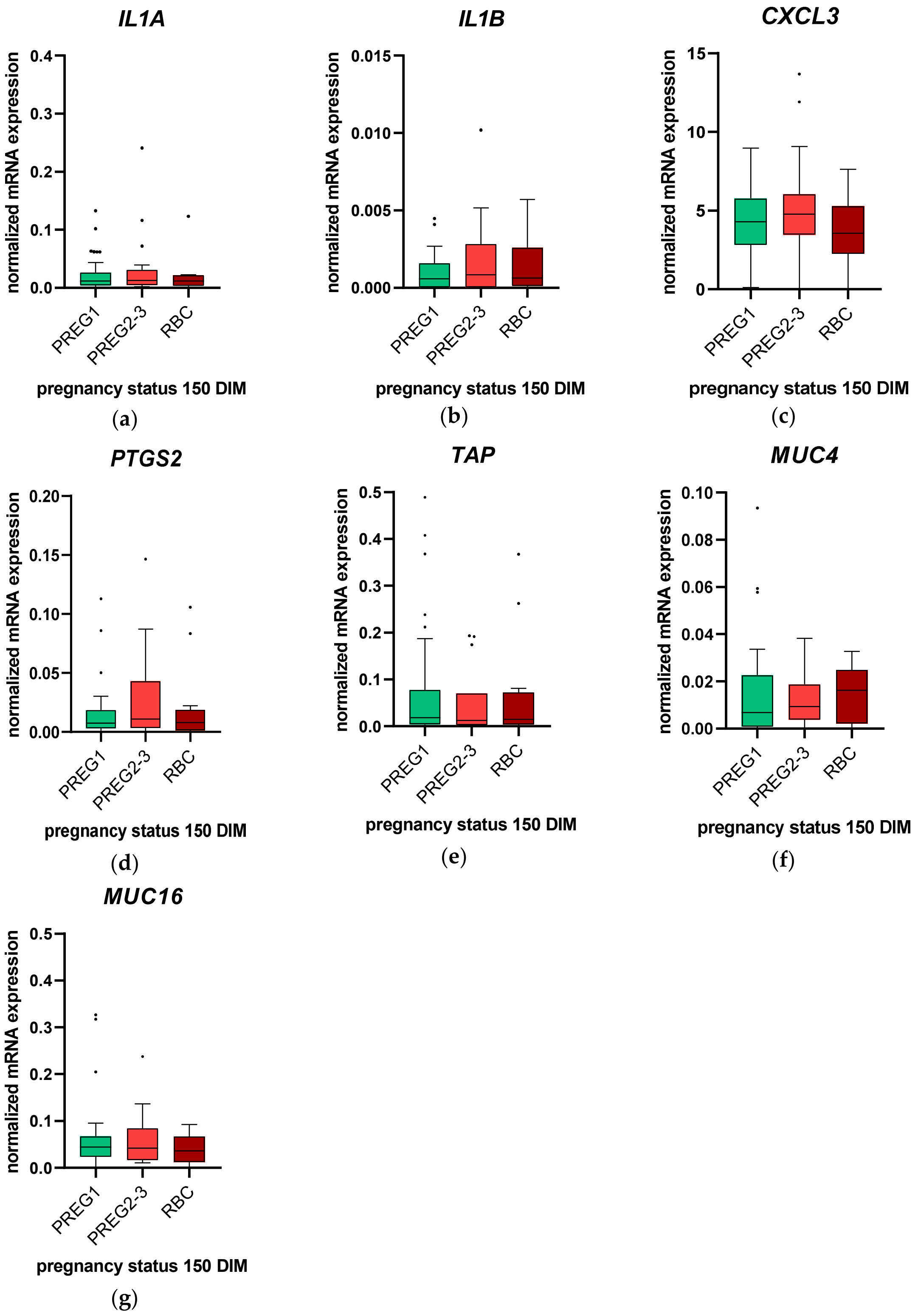

Figure S2. Normalized *IL1A* (**a**), *IL1B* (**b**) and *CXCL3* (**c**), *PTGS2* (**d**), *TAP* (**e**), *MUC4* (**f**), *MUC16* (**g**) mRNA expression in endometrial cytobrush samples collected from animals at the time of artificial insemination. Animals were retrospectively grouped in animals that conceived after the first (PREG1, $n = 32$), second to third (PREG2-3, $n = 19$) or animals with more than three unsuccessful AIs, i.e. repeat breeder cows (RBC, $n = 13$), until 150 days in milk (DIM). Values are represented as box and whisker plots with median values and 50% of the data within the boxes. Whiskers include all data points within 1.5 times the interquartile range (IQR). All outliers (≥1.5 times IQR) were included in the statistical analyses.
